# Circular RNA-ITCH Suppresses Lung Cancer Proliferation via Inhibiting the Wnt/*β*-Catenin Pathway

**DOI:** 10.1155/2016/1579490

**Published:** 2016-08-24

**Authors:** Li Wan, Lin Zhang, Kai Fan, Zai-Xing Cheng, Quan-Chao Sun, Jian-Jun Wang

**Affiliations:** Department of Thoracis Surgery, Union Hospital, Tongji Medical College, Huazhong University of Science and Technology, Wuhan 430022, China

## Abstract

As a special form of noncoding RNAs, circular RNAs (circRNAs) played important roles in regulating cancer progression mainly by functioning as miRNA sponge. While the function of circular RNA-ITCH (*cir-ITCH*) in lung cancer is still less reported, in this study, we firstly detected the expression of* cir-ITCH* in tumor tissues and paired adjacent noncancer tissues of 78 patients with lung cancer using a TaqMan-based quantitative real-time PCR (qRT-PCR). The results showed that the expression of* cir-ITCH* was significantly decreased in lung cancer tissues. In cellular studies,* cir-ITCH* was also enhanced in different lung cancer cell lines, A549 and NIC-H460. Ectopic expression of* cir-ITCH* markedly elevated its parental cancer-suppressive gene, ITCH, expression and inhibited proliferation of lung cancer cells. Molecular analysis further revealed that* cir-ITCH* acted as sponge of oncogenic miR-7 and miR-214 to enhance ITCH expression and thus suppressed the activation of Wnt/*β*-catenin signaling. Altogether, our results suggested that* cir-ITCH* may play an inhibitory role in lung cancer progression by enhancing its parental gene, ITCH, expression.

## 1. Introduction

Lung cancer is the most common incident cancer and the leading cause of cancer-related death in China [1]. Although continuous efforts have been devoted to improving the therapeutic response and treatments for stage I lung cancer have demonstrated survival benefits [2, 3], the overall five-year survival rate of advanced lung cancer is still less than 15% [4–6]. Therefore, the development of finding novel therapeutic targets is of particular importance for the treatments of lung cancers, and a further understanding of the molecular mechanisms underlying lung cancer is essential to achieve this goal.

Circular RNAs (circRNAs) represent a large class of endogenous RNAs with covalently closed continuous loop [7]. For decades, circRNAs were mostly misinterpreted as splicing errors that result from splicing artefacts or gene rearrangements [8]. But recently (from 2012/2013), circRNAs were rediscovered from RNA sequencing (RNA-seq) data and shown to be ubiquitous in mammalian cells and more abundant (certain circRNAs are up to 200 times) than their linear counterparts [9, 10]. Tissue, as well as development-specific expression of circRNAs, further indicates that they originate from nonrandom back-splice events [7, 11]. With regard to their function, several studies reported that circRNAs mainly serve as miRNA sponges to regulate gene expression [7, 12]. For at least one specific circRNA, ciRS-7, which harbors more than 70 conventional miR-7 binding sites, impairs the regulatory effect of miR-7* in vivo* [12]. miRNAs regulate a variety of essential biological functions such as cellular differentiation, apoptosis, and proliferation and thus play critical role in cancer progression [13]. Based on these clues, circRNAs were found to be closely related to development of different cancers, including esophageal squamous cell carcinoma, colorectal cancer, gastric cancer, and ovarian cancer [14–17]. Specifically, Hsa_circ_002059 expression levels are significantly correlated with distal metastasis and TNM stage of gastric cancer and thus may be a potential novel and stable biomarker for the clinical diagnosis of gastric cancer [17].

Aberrant activation of the Wnt/*β*-catenin pathway plays a critical role in tumor initiation, progression, and metastasis of lung cancer [18–20]. The E3 ubiquitin (Ub) protein ligase (ITCH) inhibits Wnt/*β*-catenin signaling in cancers mainly by promoting the ubiquitination and degradation of phosphorylated disheveled 2 (Dvl2) [21]. Circular RNA-ITCH (*cir-ITCH*) shared some miRNAs binding sites with the 3′-untranslated region (UTR) of ITCH, including those for miR-7, miR-17, miR-214, miR-128, and miR-216b [7, 12, 15]. As sponge of oncogenic miR-7, miR-17, and miR-214,* cir-ITCH* increases the level of ITCH and thus indirectly inhibits the activation of Wnt/*β*-catenin pathway; these effects finally result in the suppression of esophageal squamous cell carcinoma [16] and colorectal cancer [15]. However, there are no reported studies on the functional roles of* cir-ITCH* in lung cancer.

As two oncogenic miRNAs, miR-7 and miR-214 are overexpressed in lung cancer cells, enhance radiotherapy response, and promote the progression of lung cancer [22, 23]. Thus, in this study, we hypothesized that* cir-ITCH* might compete with ITCH to bind to miR-7 and miR-214 and may be involved in lung cancer development. To address this hypothesis, we detected the expression of* cir-ITCH* in primary tumor tissues and different lung cancer cell lines. Then, the functional relevance of* cir-ITCH* with lung cancer was further examined by biochemical assays.

## 2. Materials and Methods

### 2.1. Participants and Tissue Samples

The study was approved by the Ethical Review Board for Research in Tongji Hospital, affiliated to Tongji Medical College of Huazhong University of Science and Technology. 78 lung cancer biopsy specimens and paired adjacent normal tissues were obtained from Department of Pathology of Tongji Hospital. Tissues were acquired and immediately stored at liquid nitrogen until use. There were no limitations on the age, sex, histology, or stage of lung cancer. The patients' characteristics were summarized in Table 1.

### 2.2. Cell Culture

Human lung cancer cell lines A549 and NCI-H460 were purchased from the Cell Bank of Type Culture Collection of the Chinese Academy of Sciences, Shanghai Institute of Cell Biology. Cells were cultured in DMEM medium (Gibco, CA, USA) and supplemented with 10% fetal bovine serum (Gibco), 2 *μ*M L-glutamine, 100 U/mL penicillin, and 100 *μ*g/mL streptomycin sulfate. Cells were incubated at 37°C in a humidified atmosphere containing 5% CO_2_.

### 2.3. Circular RNA Plasmid Construction

Human* cir-ITCH* cDNA was synthesized by GeneWiz (Suzhou, China) and cloned into pcDNA3.1 (Invitrogen, CA, USA) as previously described [15, 16]. Recombinant plasmid pcDNA3.1-*cir-ITCH* was verified by direct sequencing.

### 2.4. RNA Extraction and Real-Time Quantitative Polymerase Chain Reaction

Total RNA was isolated from cells and tissues using the TRIzol reagent (Invitrogen) according to the manufacturer's instructions. RNA was reversely transcribed into cDNA using First Strand cDNA Synthesis Kit (Toyobo). The relative gene expression of* cir-ITCH* was quantified using a real-time RT-PCR with the TaqMan probe. GAPDH was used as an internal control [24], and all reactions were performed in triplicate. The primers used for polymerase chain reaction (PCR) amplification are listed in Table 2.

### 2.5. RNase R Digestion

The RNase R digestion reaction was performed following previously published procedures. The digestion and precipitation reactions were repeated twice with a ratio of 3 U of enzyme/1 mg of RNA [25].

### 2.6. Transient Transfections and Luciferase Assays

A549 and NCI-H460 cells were seeded in 24-well plates (1 × 10^5^ cells per well) and cultured to about 70% confluence before transfection. Then, cells were transfected with 800 ng of the reporter plasmids described above using Lipofectamine 2000 (Invitrogen). Cells were cotransfected with the miRNAs according to the manufacturer's instructions [26]. Each group included 6 replicates, and triplicate independent experiments were performed. 24 h after transfection, the cells were collected using 100 *μ*L passive buffer, and Renilla luciferase activity was detected using the Dual-Luciferase Reporter Assay System (Promega); the results were normalized against the activity of the Renilla luciferase gene [16].

### 2.7. Actinomycin D Assay

A549 and NCI-H460 cells were seeded at 5 × 10^4^ cells per well in 24-well plate overnight and then transiently transfected with 1 or 40 pmol of miRNA mimics (Ambion) using Lipofectamine 2000 with or without 40 pmol of miRNA inhibitor as indicated. 24 h later, cells were then exposed to 2 mg/L actinomycin D (Sigma) for 1, 2, and 3 h. The cells were harvested and the stability of the* cir-ITCH* mRNA was analyzed using quantitative reverse transcription PCR (qRT-PCR).

### 2.8. Western Blotting

Protein was isolated from cell lysis using Mammalian Protein Extraction Reagent (Thermo Fisher Scientific, Rockville, MD, USA). Equivalent amount of protein was loaded on 10% SDS-PAGE gel (Invitrogen) and then transferred onto polyvinylidene difluoride (PVDF) membranes (Millipore, Billerica, MA, USA). The PVDF membranes were blocked with 5% nonfat milk for 1 hour at 37°C. Membranes were incubated overnight at 4°C with anti-Wnt3a antibody (1 : 1000 dilution, Santa Cruz Biotechnology, Santa Cruz, CA, USA), anti-*β*-catenin antibody (1 : 1000 dilution, Santa Cruz Biotechnology), or *β*-actin (1 : 5000 dilution, Abcam, Cambridge, MA, USA) and then incubated with secondary HRP-goat anti-rabbit/mouse antibodies (1 : 10000 dilution, Santa Cruz Biotechnology). Signals were detected using ECL detection reagent (Millipore) following the manufacturer's instructions.

### 2.9. Cell Viability Assay

Cell viability assay was carried out with a Cell Counting Kit-8 (Beyotime, Shanghai, China) according to the manufacturer's instructions [27]. 1 × 10^4^ cells in 100 *μ*L of A549 and NCI-H460 cells were seeded into 96-well plates (BD Biosciences), respectively. 24 h after transfection, cells were incubated for another 1, 2, and 3 days. The numbers of cells per well were detected by the absorbance (450 nm) of reduced WST-8 at the indicated time points. The absorbance (450 nm) was measured by using SpectraMax® i3x microplate reader (Molecular Devices, Sunnyvale, CA, USA). There were 6 replicates for each group, and the experiments were repeated at least 3 times.

### 2.10. Statistical Analysis

All data are presented as mean ± SD and analyzed by using the GraphPad Prism version 5.00 software (GraphPad Software, CA, USA). Spearman correlation test was used to assess the association between* cir-ITCH* expression and the mRNA expression of ITCH in lung cancer tissues. Comparison between two groups for statistical significance was performed with two-tailed Student's *t*-test. For more groups, one-way ANOVA followed by Newman-Keuls post hoc test was used. *p* < 0.05 was considered statistically significant.

## 3. Results

### 3.1. Identification of the Circular RNA

**Figure 1:**
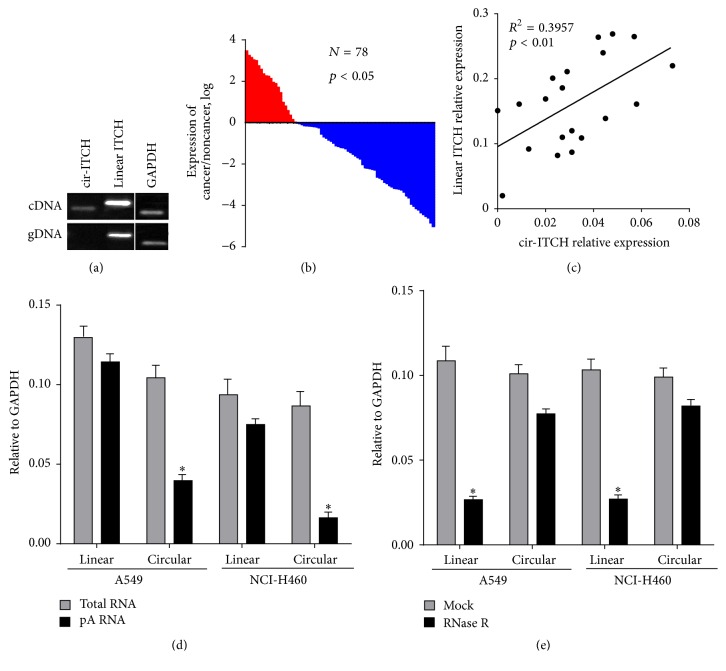
The expression level of* cir-ITCH* is closely related to lung cancer. (a)* cir-ITCH* was amplified by RT-PCR with divergent primers in cDNA but was not amplified in genomic DNA (gDNA). GAPDH, linear control. (b) qRT-PCR based on TaqMan probe was used to analyze the expression level of* cir-ITCH *in lung cancer tissues and paired noncancerous tissues. GAPDH was used as endogenous control. (c) The linear correlations between the* cir-ITCH *expression levels and linear ITCH were tested by Spearman analysis. The relative expression value was normalized by GAPDH expression level. (d) Random primers and oligo dT primers were used, respectively, in the reverse transcription experiments. The predicted circular RNA is absent in poly(A)-enriched samples. (e) The predicted circular RNA is resistant to RNase R treatment. Data are presented as mean ± SEM from three independent experiments. ^*∗*^ *p* < 0.05.

We designed two sets of primers for ITCH detection: a divergent set that was expected to amplify only the circular form and an opposite-directed set to amplify the linear forms. cDNA and genomic DNA were used as templates. The circular form, namely,* cir-ITCH*, was amplified by using the divergent primers on cDNA (Figure 1(a), upper panel). And as expected, there was no amplification when performing RT-PCR with the divergent primers on genomic DNA (Figure 1(a), lower panel). Linear-ITCH was amplificated from both of cDNA and genomic DNA templates (Figure 1(a)). GAPDH was used as a linear control (Figure 1(a)). Thus, we confirmed that* cir-ITCH* is specifically amplified with divergent primers on cDNA.

### 3.2. *cir-ITCH* Is Overexpressed and Positively Correlated with ITCH Expression in Lung Cancer Tissues

Next, cDNA of cancer tissues and paired noncancerous tissues of 78 lung cancer patients was extracted, and then the expression level of* cir-ITCH* was evaluated with the divergent primer set.* cir-ITCH* was expressed at a lower level in approximately 73.08% of the lung cancer tissues compared to that of the paired noncancerous samples (Figure 1(b)). Simultaneously, we further evaluated the association between* cir-ITCH* expression in lung cancer tissues and clinical characteristics of lung cancer patients. As shown in Table 1,* cir-ITCH* expression in lung cancer tissues was significantly associated with age (*p* = 0.0076); however, it is not correlated with other clinical characteristics including sex (*p* = 0.9704), family history (*p* = 0.8437), smoking (*p* = 0.1713), drinking (*p* = 0.1568), tumor type (*p* = 0.9183), and TNM stage (*p* = 0.2531) in lung cancer patients. To study the correlation between* cir-ITCH* and ITCH in lung cancer, we assessed the expression of* cir-ITCH* in randomly selected 20 pairs of tissue samples from the 78 patients. The results showed that patients with higher* cir-ITCH* expression levels in lung cancer tissues had a substantial upregulation of linear ITCH (*R* ^2^ = 0.33, *p* < 0.01; Figure 1(c)).

### 3.3. Characterization of* cir-ITCH* in Lung Cancer Cells

To further study the role of* cir-ITCH* in lung cancer progression, we constructed a recombinant vector to express* cir-ITCH *in lung cancer cell lines according to the previous studies [15, 16]. Then, the constructed plasmid was transiently transfected into A549 and NCI-H460 cells. Next, random primers and oligo (dT) primers were used to reverse total RNA and mRNA into cDNA, respectively. In contrast to the linear products, we thought that circular products amplified with the divergent primer set would be depleted in the poly(A)-enriched RNA [28]. In our results, the expression of linear ITCH (normalized to GAPDH) showed no difference between total RNA and poly(A)-enriched RNA in both A549 cells and NCI-H460 cells (Figure 1(d)), while the expression of* cir-ITCH* was significantly decreased in poly(A)-enriched RNA compared with total RNA in these two lung cancer cell lines (Figure 1(d)).

Circular RNAs are resistant toward exonucleases for the reason of lacking free ends [29, 30]. To further confirm the circular characteristics of* cir-ITCH* in A549 cells and NCI-H460 cells, the enzyme RNase R, a highly processive 3′ to 5′ exoribonuclease that does not react on circular RNAs, was used to digest total RNA [31, 32], and then we performed RT-PCR to evaluate linear ITCH and* cir-ITCH* expression. As expected, in contrast to the linear ITCH, the predicted* cir-ITCH* was resistant to the RNase R treatment (Figure 1(e)).

### 3.4. Interaction between* cir-ITCH* and miRNA

**Figure 2:**
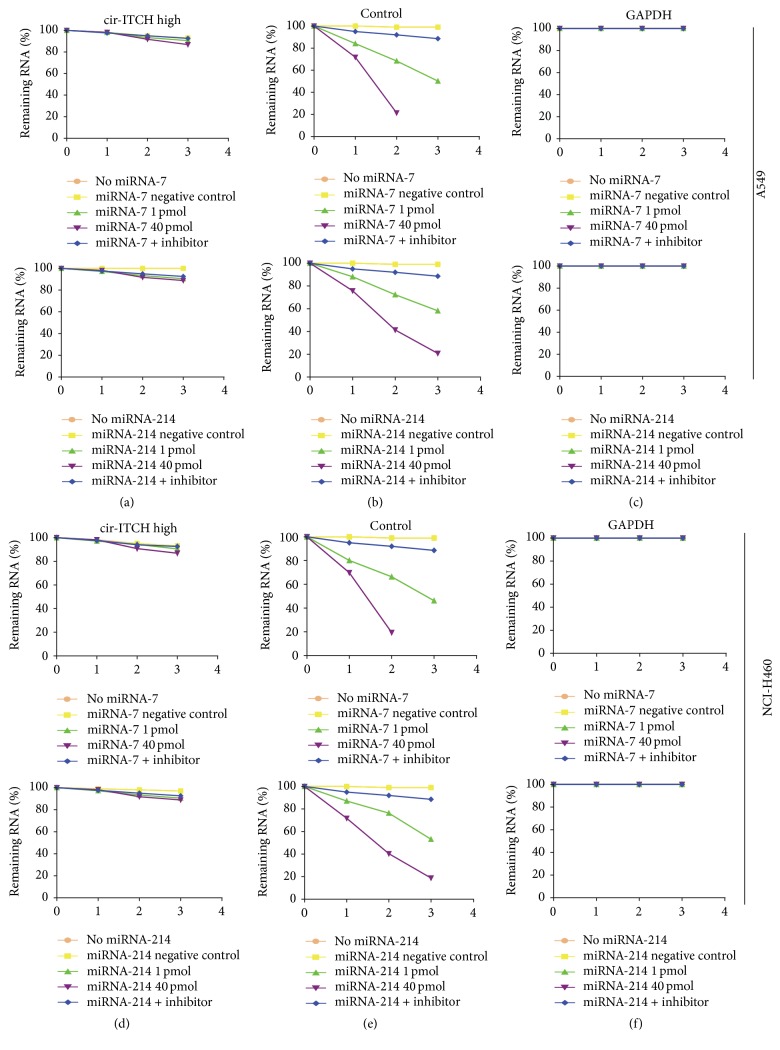
*cir-ITCH* inhibits the expression of miR-7 and miR-214. A549 cells were, respectively, transfected with* cir-ITCH* (a) and empty control vector (b) and simultaneously treated with miR-7 (upper) and miR-214 (lower) for 24 h. Cells were then further exposed to actinomycin D for 1, 2, and 3 h. The stability of* cir-ITCH* mRNA was analyzed by qRT-PCR relative to 0 h after actinomycin D treatment. (c) GAPDH as endogenous control. ((d)–(f)) Similar to A549 cells, the stability of* cir-ITCH* mRNA in NCI-H460 cells was evaluated by qRT-PCR. Data are presented as mean ± SEM, normalized to GAPDH.

miR-7 and miR-214 can bind to the 3′-untranslated region (UTR) of ITCH and* cir-ITCH* [16], and binding sites of these two miRNAs were presented in Table 3. The stability of* cir-ITCH* was firstly investigated with the presence of miRNA mimic or inhibitor via actinomycin D assay. A549 and NCI-H460 cells were cotransfected with the* cir-ITCH* plasmid and miRNA mimic or inhibitor, respectively. And, then, cells were treated with actinomycin D, a transcription inhibitor. Total RNA was extracted at indicated time points and the relative expression of* cir-ITCH *was evaluated. There was almost no change in* cir-ITCH* levels in both A549 cells and NCI-H460 cells with miRNA mimic or inhibitor treatment (Figures 2(a) and 2(d)), while the* cir-ITCH* levels in cells transfected with empty vector remained only 20–30% (Figures 2(b) and 2(e)), which were significantly lower than that in cells transfected with the* cir-ITCH* plasmid (*p* < 0.01). These results suggest that miR-7 and miR-214 can degrade* cir-ITCH *in lung cancer cells.

**Figure 3:**
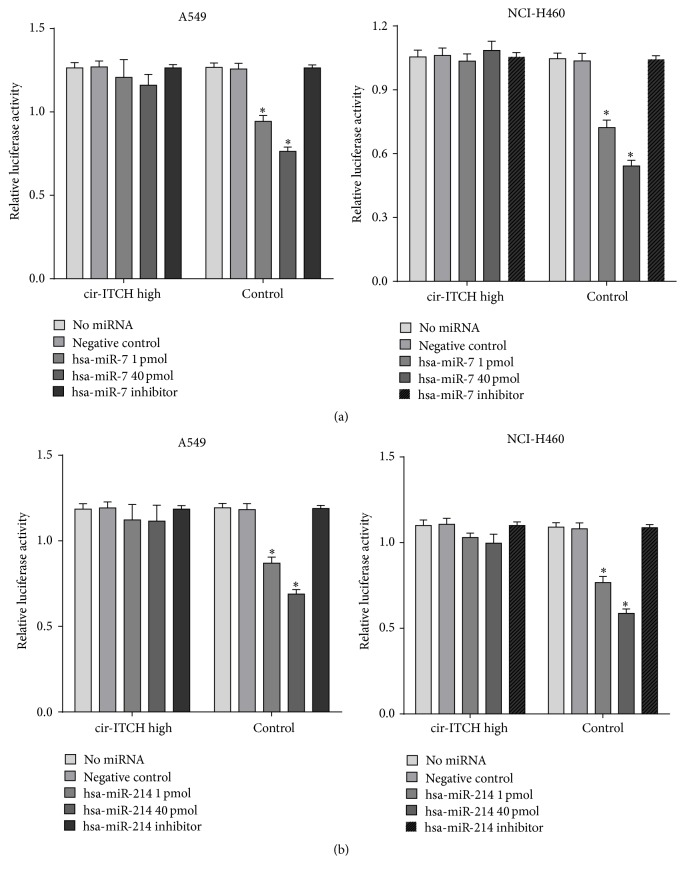
*cir-ITCH* acts as microRNA sponges of miR-7 and miR-214. (a) A549 (left) and NCI-H460 (right) cells were cotransfected with psiCHECK-2-ITCH constructs and* cir-ITCH* or empty control vector, respectively. Cells were further simultaneously treated with miR-7 mimic or inhibitor for 24 h. Then, relative luciferase activity of psiCHECK-2-ITCH constructs was evaluated. (b) Similar to miR-7, relative luciferase activity of the psiCHECK-2-ITCH constructs in A549 and NCI-H460 cells with the presence of miR-214 was evaluated. Data are presented as mean ± SEM from six replicates for each group. ^*∗*^ *p* < 0.05.

Next, the ITCH binding sequences of miR-7 and miR-214 were inserted into psiCHECK-2 vector, respectively. The constructed luciferase reporter of miR-7 or miR-214 and* cir-ITCH* plasmid were transiently cotransfected into lung cancer cells, and the luciferase activity was subsequently detected. In both A549 cells (Figure 3(a)) and NCI-H460 cells (Figure 3(b)) transfected with empty vector (control of* cir-ITCH* plasmid), luciferase activity was significantly decreased in a concentration-dependent manner with the presence of miR-7/miR-214 mimic. However, there were no significant differences in luciferase activity of miR-7/miR-214 mimic in cells with* cir-ITCH* hyperexpression (Figures 3(a) and 3(b)). Thus,* cir-ITCH *can act as sponge of ITCH to interact with miR-7 and miR-214 in lung cancer cells.

### 3.5. *cir-ITCH* Inhibits the Activation of Wnt/*β*-Catenin Signaling Pathway

**Figure 4:**
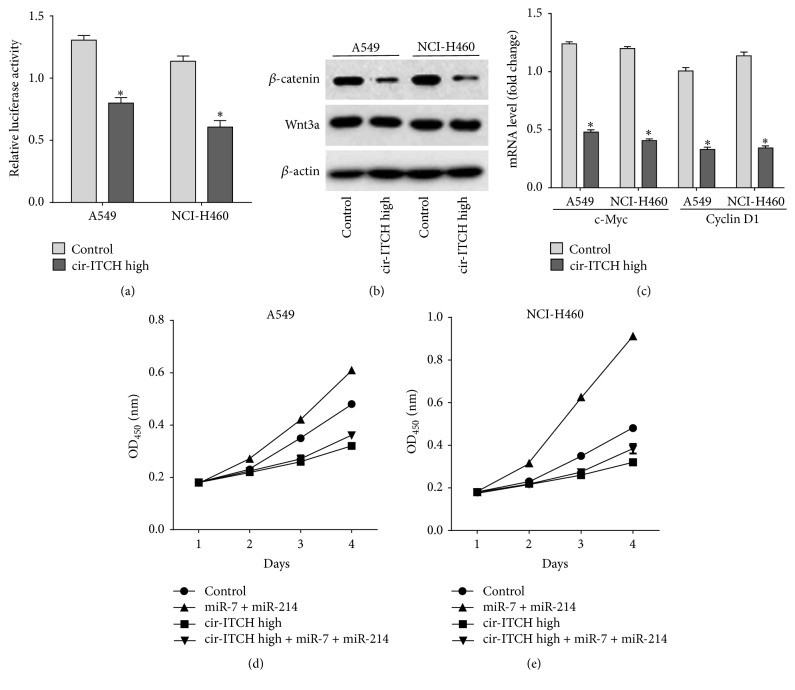
*cir-ITCH* can regulate the Wnt/*β*-catenin signaling pathway and cellular proliferation of lung cancer cells. (a) A549 and NCI-H460 cells were transfected with* cir-ITCH* or empty control vector; then, a *β*-catenin/T-cell factor- (TCF-) responsive luciferase reporter assay was performed. The luciferase activity was normalized to the Renilla luciferase activity. (b) The protein levels of Wnt3a and *β*-catenin were assessed in A549 cells and NCI-H460 cells by western blotting. *β*-actin was used as endogenous control. (c) The mRNA level of c-Myc and cyclinD1 in A549 cells and NCI-H460 cells was detected by qRT-PCR after being transfected with* cir-ITCH* or empty control vector. (d) A549 and (e) NCI-H460 cells were seeded in 96-well plates after being transfected with* cir-ITCH* and empty control vector, and cell proliferation was detected daily for 3 days by using the CCK-8 assay. Data are presented as mean ± SEM from six replicates for each group. ^*∗*^ *p* < 0.05.

ITCH protein promotes the degradation of phosphorylated Dvl2, which is an important regulator for Wnt/*β*-catenin signaling activation [21].* cir-ITCH*, acting as a sponge of oncogenic miRNAs, can competitively inhibit these miRNAs' bind to ITCH and thus indirectly suppresses the activation of Wnt/*β*-catenin signaling in esophageal squamous cell carcinoma [16]. To further confirm whether* cir-ITCH* regulates the Wnt/*β*-catenin signaling pathway in lung cancer cells, we used a *β*-catenin/T-cell factor- (TCF-) responsive luciferase reporter assay [33]. As shown in Figure 4(a), overexpression of* cir-ITCH* significantly suppressed relative TCF transcriptional activity in both A549 cells and NCI-H460 cells, which suggests that* cir-ITCH* inhibits *β*-catenin expression. The expression level of *β*-catenin in lung cancer cells with* cir-ITCH* hyperexpression was further confirmed by western blotting analysis (Figure 4(b)), and it was discovered that there was an obvious decrease in *β*-catenin levels, while no change in Wnt3a expression was shown. Oncogene c-Myc and cell cycle regulator cyclinD1 are two important downstream targets of *β*-catenin [33, 34]; then, we investigated the effect of* cir-ITCH* on the mRNA expression of these two proteins. In lung cancer cells transfected with* cir-ITCH*, mRNA expression of c-Myc and cyclinD1 was significantly suppressed compared to empty vector control (Figure 4(c)).

### 3.6. *cir-ITCH* Inhibits Cellular Proliferation of Lung Cancer

*cir-ITCH* inhibits cell proliferation in both esophageal squamous cell carcinoma and colorectal cancer [15, 16]. To further confirm the role of* cir-ITCH *in lung cancer cell proliferation, lung cancer cells were transfected with* cir-ITCH *with or without miR-7 and miR-214. We noticed that miR-7 and miR-214 significantly promoted cell proliferation of A549 cells and NCI-H460 cells compared to simple empty vector transfection, while cell proliferation was dramatically decreased when* cir-ITCH* was overexpressed with the presence of miR-7 and miR-214 treatment compared to the controls. In lung cancer cells transfected with* cir-ITCH *with or without miR-7 and miR-214, treatment showed no significant difference (Figures 4(d) and 4(e)).

## 4. Discussion

Recently, many studies have confirmed the widespread and abundant presence of circular RNA in eukaryotic cells [35–38]. Specifically,* cir-ITCH *has been reported to inhibit the progression of esophageal squamous cell carcinoma and colorectal cancer mainly by regulating the Wnt/*β*-catenin pathway [15, 16]. However, the function of* cir-ITCH* in lung cancer is still unclear. In this study, we compared the expression level of* cir-ITCH* in lung cancer issues by using a TaqMan-based RT-PCR and found that* cir-ITCH *was dramatically decreased in lung cancer tissues, indicating* cir-ITCH* may play a role in regulating lung cancer progression.

*cir-ITCH*, located on chromosome 20q11.22, is aligned in a sense orientation to the known protein-coding gene, ITCH, a member of the E3 ubiquitin ligases [16]. Correlation analysis showed that* cir-ITCH* expression in lung cancer tissues was not correlated with clinicopathological characteristics except age. This is likely to be because the incidence of lung cancer increases with age, particularly after the age of 60 [39], and we still need to confirm this result in a larger sample population. As parental gene of* cir-ITCH*, ITCH also decreased in lung cancer patients and positively correlated with* cir-ITCH*. Hence, we speculated that ITCH may play a tumor suppressive role in lung cancer, and there is a possible connection between ITCH and* cir-ITCH*. ITCH involved in cancer progression mainly depends on its ability to regulate protein stability [40–42] and the target proteins including p63 [43], p73 [44], Notch1 [45], Dvl2 [21], RASSF5 [42], and LATS1 [41]. These proteins usually associated with tumor formation and chemosensitivity serve as either tumor suppressor or enhancer; thus, the role of ITCH in tumor progression is complicated.

Concerning the function of* cir-ITCH*, it is speculated that* cir-ITCH* serves as epigenetic miRNA sponges to competitively block the bind between miRNA and ITCH. Previous research has shown that miR-216b, miR-17, miR-214, miR-7, miR-20a, and miR-128 could bind to the 3′-UTR of ITCH and cir-ITCH [15, 16]. In our study, we found that miR-7, miR-214, and miR-128 (data not shown) decreased ITCH expression by binding to its 3′-UTR in lung cancer cell lines, and* cir-ITCH* acts as a sponge for miR-7 and miR-214, except miR-128. These results were not fully consistent with study in cell lines of esophageal squamous cell carcinoma [16], in which* cir-ITCH *acts as a sponge for five miRNAs: miR-216b, miR-17, miR-214, miR-7, and miR-128. The reason may be owing to the difference of cancer origin, as the expression and function of circRNAs occupy tissue- and development-specific properties [11].* cir-ITCH* also did not act as a sponge for miR-214 but for miR-20a in colorectal cancer [15]. As two oncogenic miRNAs, miR-7 and miR214 are involved in the progression of many cancers including lung cancer [22, 23, 46–49]. In lung cancer cells, we found that miR-7 and miR214 promoted cell proliferation; this activity was totally abrogated with ectopic* cir-ITCH *hyperexpression. Thus,* cir-ITCH* is involved in lung cancer progression by interacting miRNAs. However, with regard to other tumor-related activities, like migration, invasion, and colony formation, the antitumor effects of* cir-ITCH* in lung cancer still need to be further investigated.

ITCH is crucial in the control of proteasome degradation of Dvl2, which inhibits Wnt/*β*-catenin signaling [21, 50]. Deregulated Wnt/*β*-catenin signaling with cancers has been well documented in tumor initiation, progression, and metastasis, including lung cancer [18–20, 51, 52]. Blocking *β*-catenin signaling for cancer treatment has thus generated significant interests [53]. The beneficial effect of nonsteroidal anti-inflammatory drugs (NSAIDs) in cancer prevention and therapy has been attributed partially to the perturbation of *β*-catenin signaling [54]. In our study,* cir-ITCH* inhibits Wnt/*β*-catenin signaling in lung cancer cells with the evidence that hyperexpression of* cir-ITCH* significantly suppressed relative TCF transcriptional activity in *β*-catenin/TCF-responsive luciferase reporter assay. This result was further confirmed by western blotting analysis.

At last, we examined the impacts of* cir-ITCH *on two important downstream targets of Wnt/*β*-catenin pathway, c-Myc and cyclinD1, which are continually overexpressed in many cancers and have crucial roles in regulating cell growth, apoptosis, and differentiation [55]. Hyperexpression of* cir-ITCH* significantly suppressed the mRNA expression of c-Myc and cyclinD1 in lung cancer cells. Combined with previous studies [15, 16], we are able to conclude that* cir-ITCH* has an antitumor role in lung cancer by controlling miRNA activity, which increases the concentration of ITCH and results in suppression of the canonical Wnt/*β*-catenin pathway.

In conclusion, our study demonstrates that the* cir-ITCH* acts as a sponge for miR-7 and miR-214, promotes the expression of their target gene ITCH, and thus regulates lung cancer cell proliferation by indirectly inhibiting the activation of Wnt/*β*-catenin pathway. Further characterization of the function of circular RNAs in cancer progression will have great implication for the development of new RNA-based cancer diagnosis and therapy.

## Figures and Tables

**Table 1:** Baseline demographic and clinical characteristics of study populations.

Characteristics	Population		cir-ITCH relative expression^#^	*p* value
	*N*	(%)		
*Age (years)*				
≤40	10	12.82	0.0607 ± 0.0169	0.0076^*∗*^
40–60	31	39.74	0.0466 ± 0.0307	
≥60	37	47.44	0.0357 ± 0.0155	

*Sex*				
Male	45	57.7	0.0432 ± 0.0257	0.9704
Female	33	42.3	0.0434 ± 0.0225	

*Family history*				
Yes	23	29.49	0.0441 ± 0.0290	0.8437
No	55	70.51	0.0429 ± 0.0221	

*Smoking*				
Never	53	67.95	0.0487 ± 0.0283	0.1713
Ever	25	32.05	0.0407 ± 0.0230	

*Drinking*				
Never	33	42.3	0.0478 ± 0.2352	0.1568
Ever	45	57.7	0.0399 ± 0.0244	

*Tumor type*				
Adenocarcinoma	29	37.18	0.0443 ± 0.0267	0.9183
Large cell carcinoma	25	32.05	0.0436 ± 0.0250	
Squamous cell carcinoma	24	30.77	0.0416 ± 0.0218	

*Stage*				
I	18	23.08	0.0605 ± 0.0273	0.0011^*∗*^
II	21	26.92	0.0444 ± 0.0260	
III	22	28.21	0.0375 ± 0.0182	
IV	17	21.79	0.0309 ± 0.0142	

^*∗*^ *p* < 0.05 means statistically significant difference existed within subgroups.

^#^Relative expression value was normalized to GAPDH expression level.

**Table 2:** The sequences of primers used in this study.

Gene	Forward (5′-3′)	Reverse (5′-3′)	Probe
cir-ITCH	GCAGAGGCCAACACTGGAA	TCCTTGAAGCTGACTACGCTGAG	CCGTCCGGAACTATGAACAACAATGGCA
GAPDH	CCATGACCCCTTCATTGACC	TTGATTTTGGAGGGATCTCG	CTGAGAACGGGAAGCTTGTC
Linear ITCH	TAGACCAGAACCTCTACCTCCTG	TTAAACTGCTGCATTGCTCCTTG	
Circular ITCH	ACAGAGACAACCGAGAAACAGTG	GCCTTGATACTTGTTACCGTCGA	
c-Myc	TTCGGGTAGTGGAAAACCAG	CAGCAGCTCGAATTTCTTCC	
cyclinD1	GAGGAGCAGCTCGCCAA	CTGTCAAGGTCCGGCCAGCG	
GAPDH	GAAGGTGAAGGTCGGAGTC	GAAGATGGTGATGGGATTTC	

**Table 3:** The sequence of the predicted miRNA binding sites on the 3′-UTR region of ITCH and *cir-ITCH*.

MicroRNA	miRNA binding sites 3′-UTR	miRNA binding sites in *cir-ITCH*
miRNA-7	GUGGCCACAUGUAUAUAGUCUUCCC	UGAGGUAGUAGGUUGUAUAGUU
miRNA-214	UGUAUAUGUCUUCCCUGCUGU	ACAGCAGGCACAGACAGGCAGU
